# The efficacy and adverse effects of PARP inhibitor combined with chemotherapy compared with chemotherapy alone in the treatment of cancer patient: A protocol for systematic review

**DOI:** 10.1097/MD.0000000000023040

**Published:** 2020-11-06

**Authors:** Suyue Zhao, Tao Fang, Li Yao, Ying Zheng, Ling Zhang, Kexiang Zhu

**Affiliations:** a The First Clinical Medical College of Lanzhou University; b The First Hospital of Lanzhou University, China.

**Keywords:** chemotherapy, neoplasms, PARP inhibitors, protocols, randomized controlled trials, systematic reviews

## Abstract

**Background:**

There search of PARP inhibitors has made great breakthroughs and progress. Become a new type of medicine for cancer treatment,bringing hope to more advanced cancer patients.The purpose of this systematic review is to evaluate the clinical efficacy and adverse effects of PARP inhibitorscombined with chemotherapy and chemotherapy alone in the treatment of cancer patients.

**Methods:**

We searched the following 4 databases, including: PubMed, EMBASE, Web of Science, and Cochrane Library. The search will also be conducted at the clinical trial centers: ClinicalTrials.gov, ISRCTN Registry, WHO International Clinical Trials Registration Platform. The search date is as of September 22, 2020. There is no language restriction during this search, and the latest documents are kept updated through settings. The subject search terms were identified as “PARP Inhibitor”, “Neoplasms” and “Dug therapy”. The Phase 2 and Phase 3 clinical trials comparing PARP inhibitor combined with chemotherapy and chemotherapy alone were included. The results include overall survival (OS), progression-free survival (PFS), objective response rate (ORR) and adverse events. Two researchers separately completed the article inclusion, data extraction and quality evaluation of this study. The assessment of the risk of bias and data will be conducted using Review Manager.

**Ethics and dissemination:**

All articles are published and do not require the approval of the ethics committee and the signed informed consent form. The results of this systematic review will be published through peer-reviewed publications.

**Registered:**

Registered on INPLASY and the registration number is INPLASY202090087.

## 1 Introduction

In the world, tumors are one of the most important diseases that increase the burden of human economy and medical care. Although with the development of medical treatment, the survival time of patients can be prolonged through various treatments such as surgery and drugs, there is no doubt that tumors are still an unsolved problem. In the past 10 years, the research of PARP inhibitors has made great breakthroughs and progress. Become a new type of medicine for cancer treatment, bringing hope to more advanced cancer patients.

DNA damage repair is essential to maintain stable genetic inheritance. When DNA is damaged in the cell, the body can repair DNA damage through specific ways, including homologous recombination repair, non-homologous end joining repair, and single strand break repair.^[1,2]^ PARP inhibitors use defects in DNA repair to induce cell death.^[3]^ PARP repairs broken DNA by binding to damaged DNA at the single-stranded DNA break site to initiate repair.^[4]^ PARP1 can repair not only broken single-stranded DNA but also double-stranded DNA. PARP2 can only repair broken single-stranded DNA.^[5]^ HR can accurately restore damaged double-stranded DNA. BRCA1/2 protein is essential for the repair of HR.^[6]^ Tumor cells with BRCA1/2 gene mutations are more sensitive to PARP inhibitors through synthetic lethal mechanisms due to DNA repair defects. Therefore, PARP inhibitors can lead to the death of cancer cells in patients with BRCA gene mutations, serving as a therapeutic purpose.^[7,8]^ Mutations in the BRCA1/2 gene are susceptible to ovarian cancer and breast cancer. About 10% of breast or ovarian cancer patients carry BRCA1/2 gene mutations. In addition, BRCA1/2 somatic mutations have also been proposed in various cancers.^[9–11]^ With the deepening of research, 4 PARP inhibitors olaparib, rucaparib, niraparib, and talazoparib have been approved by the Food AND Drug Administration (FDA) and the European Medicines Agency (EMA).^[5]^ From 2014 to 2016, olaparib and rucaparib were approved to treat advanced ovarian cancer. From 2017 to 2018, olaparib, rucaparib, and niraparib were approved for the treatment of ovarian cancer, fallopian tube cancer, and primary peritoneal cancer. Through a number of large-scale clinical trials, the safety and efficacy of PARP inhibitors are widely recognized. The latest meta-analysis based on a number of large clinical trials shows that PARP inhibitors can significantly prolong the survival of cancer patients. And PARP inhibitors are more sensitive to the treatment of ovarian cancer patients with BRCA1/2 mutations. Research results show that PARP inhibitors have curative effects on prostate cancer, pancreatic cancer, and small cell lung cancer.^[12]^ In a meta-analysis of platinum-sensitive recurrent ovarian cancer by Tomao et al, it was shown that PARP inhibitors are effective for patients regardless of BRCA mutation status.^[13]^

PARP inhibitor monotherapy has achieved great success, but PARP combination therapy is unknown. PARP inhibitors in combination with chemotherapy, antiangiogenic agents, and immunotherapy have a positive or negative effect on treatment. Which type of combination can give patients the greatest benefit and the least side effects is still a question we need to explore.

### 1.1 Review question

1. How much benefit does PARP inhibitor combined with chemotherapy bring to patients compared with chemotherapy alone?
2. Is it safe to use PARP inhibitors in combination with chemotherapy and what are the main adverse events?

### 1.2 Objective

Our purpose is mainly to evaluate PARP inhibitors in combination with chemotherapy and chemotherapy alone in cancer patients. In this research, we will address the following questions.

1. Determine the overall benefits of PARP inhibitors combined with chemotherapy compared with chemotherapy alone in all type of cancer.
2. Determine which type of combination therapy can bring the most benefits
3. Determine the adverse effects of PARP inhibitors combined with chemotherapy and chemotherapy alone in all types of tumors

## 2 Method and design

The reporting method of the experiment in this research is conducted in accordance with the Guidelines for Systematic Review and Statement of Meta-Analysis and has been registered INPLASY.^[14]^ The registration number is INPLASY202090087 and DOI number is 10.37766/inplasy2020.9.0087. Our research team is composed of clinicians and statisticians, all of whom contributed to this research.

### 2.1 Inclusion criteria for this review

#### 2.1.1 Type of participants

All cancer patients are included in this study, without restrictions on country, gender, age, race, and tumor type.

#### 2.1.2 Type of studies

We included phase 2 and phase 3 clinical trials comparing PARP inhibitor combined with chemotherapy and chemotherapy alone. There are no language restrictions on the study. Non-RCT research will be excluded.

#### 2.1.3 Intervention measures

Cancer patients are included in clinical trials. The experimental group is PARP inhibitor combined with chemotherapy and the control group is chemotherapy alone. Regardless of whether the treatment is first-line treatment, regardless of the order of administration and dosage changes, regardless of whether the patient is an advanced cancer patient. Exclude PARP inhibition combined with other non-chemotherapy experiments

#### 2.1.4 Outcome

Outcome indicators include OS, PFS, ORR and adverse effects. The adverse effects include: abdominal pain, constipation, diarrhea, fatigue, nausea, vomiting, loss of appetite, anemia, neutropenia, and thrombocytopenia.

### 2.2 Search strategy

We searched the following 4 databases, including: PubMed, EMBASE, Web of Science and Cochrane Library. The search will also be carried out at clinical trial centers: ClinicalTrials.gov, ISRCTN Registry, WHO International Clinical Trials Registration Platform. We will also search for all relevant articles including systematic reviews and literature reviews to prevent the omission of any eligible studies. The search date is as of September 22, 2020. There is no language restriction during this search. Select subject words and free words for search. The subject words are “PARP Inhibitor”, “Neoplasms”, and “Dug therapy”. Free words are Inhibitors of Poly(ADP-ribose) Polymerases, Poly(ADP-ribosylation) Inhibitors, PARP Inhibitors, Inhibitors, PARP, Neoplasia, Neoplasias, Neoplasm, Tumors, Tumor, Cancer, Cancers, Malignancy, Malignancies, Malignant Neoplasms, Malignant Neoplasm, Neoplasm, Malignant, Neoplasms, Malignant, Benign Neoplasms, Neoplasms, Benign, Benign Neoplasm, Neoplasm, Benign, Therapy, Drug, Drug Therapies, Therapies, Drug, Chemotherapy, Chemotherapies, Pharmacotherapy, Pharmacotherapies. Search strategy is shown in Table 1.

**Table 1:** Search strategy used in PubMed database.

Search	Search term
1	“PARP Inhibitor”[Mesh]
2	Inhibitors of Poly(ADP-ribose) Polymerases OR Poly(ADP-ribosylation) Inhibitors OR PARP Inhibitors OR Inhibitors, PARP
3	1 OR 2
4	“Neoplasms”[Mesh]
5	Neoplasia OR Neoplasias OR Neoplasm OR Tumors OR Tumor OR Cancer OR Cancers OR Malignancy OR Malignancies OR Malignant Neoplasms OR Malignant Neoplasm OR Neoplasm,Malignant OR Neoplasms, Malignant OR Benign Neoplasms OR Neoplasms, Benign OR Benign Neoplasm OR Neoplasm, Benign.
6	4 OR 5
7	“Dug therapy”[Mesh]
8	Therapy, Drug OR Drug Therapies OR Therapies, Drug OR Chemotherapy OR Chemotherapies OR Pharmacotherapy OR Pharmacotherapies
9	7 OR 8
10	3 AND 6 AND 9

### 2.3 Data collection and analysis

#### 2.3.1 Studies selection

The titles and abstracts of all retrieved documents are independently read by 2 researchers to exclude obviously irrelevant documents. During the screening process, documents found relevant to the research are read in full text. The inclusion and exclusion of literature are strictly based on the inclusion and exclusion criteria. The 2 researchers will be reviewed by a third party and discussed and resolved for any disputes. The excluded studies will be listed in the table with the reasons for the exclusion. Inclusion criteria:

1. Literature on complete randomized clinical trials of PARP inhibitors combined with chemotherapy.
2. The included research items include phase II and phase III clinical trials.
3. The included studies mentioned OS or PFS.

Exclusion criteria:

1. Non-randomized controlled experiment.
2. Unable to extract research data.
3. Exclude PARP inhibition combined with other non-chemotherapy experiments
4. If the publication is repeated or the publication is constantly updated, the latest article will be used in this study.

#### 2.3.2 Data extraction

We created a standard data extraction form and conducted data extraction training for data extraction researchers. Two researchers independently extracted data. If you find any data that cannot be extracted in the literature, contact the corresponding author of the article by email. Any problems during the data extraction process will be resolved by consulting a third party. The content of the extracted data includes: the name of the first author, research type, publication year, research design method, tumor type, all sample size of the experiment, separate sample size of intervention group and control group, treatment and dosing plan, baseline characteristics, clinical outcome indicators, the number of related adverse reactions. When multiple articles describe the same clinical trial, only the latest research report is included.

#### 2.3.3 Assessment of risk of bias

We use the Cochrane Intervention Systematic Review Manual, and will follow the guidance in the latest edition of the Cochrane Manual to systematically review the included literature.^[15,16]^ This process is completed by 2 researchers individually, and a third party resolved any disagreements. The researchers evaluated the risks according to the following criteria, and the risks are divided into 3 levels: “high”, “low” or “unclear”. The evaluation criteria are shown in Table 2.

**Table 2:** The Cochrane Collaboration's biased risk assessment tool.

Evaluation item	Evaluation content and description
1. Random allocation method	Describe the method of generating random assignments, which can help assess comparability between groups
2. The allocation plan is hidden	Describe the method of hiding the random allocation sequence to help determine whether the allocation of interventions is predictable
3. Blind method	Describe the method of blinding subjects and researchers, whether there are blind methods for evaluating participants, personnel, and results
4. The integrity of the data	Whether to report the completeness of the data for each outcome indicator, including those who were lost to follow-up and withdraw, and the reason for withdrawal.
5. Reporting bias	Whether to report results and data selectively
6. Other biases	Except for the above 5 categories, whether there are other risks of bias

#### 2.3.4 Outcome indicators

The main outcomes of this meta-analysis were OS and PFS. We used hazard ratio (HR) and 95% confidence interval (95% Cl) to compare OS and PFS. The risk ratio (RR) of the 95% confidence interval is used to measure the ORR of the 2 groups. Adverse events are binary data based on OR and 95% Cl. The results analysis of each study are graphically represented using forest maps. *P* < .05 was considered statistically significant.

### 2.4 Data analysis

#### 2.4.1 Strategy for date synthesis

A meta-analysis of the data is performed after the data in the included literature is extracted. RevMan 5.3 is used for data analysis. In the random effects model, we merged the dichotomous data into OR, and the time-time secretary is aggregated into HR.HR is directly extracted from the article or extracted by analyzing the survival curve. Since this study included different types of tumors, random effects models were used to reduce heterogeneity and increase reliability. *P* < .05 was considered statistically significant, and was measured using the *I*^2^-test.

#### 2.4.2 Subgroup analysis and sensitivity analysis

We use subgroup analysis to analyze the causes of heterogeneity. It is possible to perform subgroup analysis for different tumor types, ages (<30, 30–60,>60 years), genders, races, and no chemotherapy regimens. Perform sensitivity analysis to resolve the impact of each study on synthesis. We also evaluate the impact of sample size and missing data on the results. The funnel chart is used to assess the risk of publication bias. We will Using Begg and Egger tests to assess publication bias.

### 2.5 Ethics and morality

We have included published literature, so it does not involve infringement of patient privacy, so there is no need for ethics committee approval and signing of informed consent. The results of this systematic review will be published through peer-reviewed publications.

## 3 Conclusion

PARP inhibitors can significantly prolong the survival time of tumor patients as new therapeutic drugs. Monotherapy has achieved great success, but PARP combination therapy is unknown. PARP inhibitor combined with chemotherapy has a positive or negative effect on the treatment. Which type of combination can give patients the greatest benefit and the least side effects? In this systematic review, we will evaluate the therapeutic efficacy of PARP inhibitors combined with chemotherapy for cancer patients. The process of conducting this review will be divided into 4 parts: determining the inclusion research criteria, data retrieval, data extraction and data analysis. The conclusions of this review provide a theoretical basis for clinicians when choosing treatment options.

## Author contributions

Suyue Zhao and Tao Fang designed a systematic review and provided research ideas. Li Yao wrote the article and Ying Zheng revised the manuscript. Ying Zheng and Ling Zhang independently screened articles, extracted data, assessed bias and processed data. During the review, any objections will be arbitrated by Kexiang Zhu. All authors agree with the publication of the proposal.

**Conceptualization:** Suyue Zhao, Tao Fang.

**Data curation:** Ying Zheng, Ling Zhang.

**Methodology:** Suyue Zhao, Tao Fang.

**Project administration:** Kexiang Zhu.

**Writing – original draft:** Li Yao, Ying Zheng.
